# Comparative Study of Intravenous Iron Versus Intravenous Ascorbic Acid for Treatment of Functional Iron Deficiency in Patients Under Hemodialysis: A Randomized Clinical Trial

**DOI:** 10.5812/numonthly.12038

**Published:** 2013-07-24

**Authors:** Omid Sedighi, Atieh Makhlough, Ghasem Janbabai, Mohammad Neemi

**Affiliations:** 1 Department of Nephrology, Imam Khomeini Hospital, Mazandaran University of Medical Sciences, Sari, IR Iran; 2 Molecular and Cell Biology Research Center, Department of Nephrology, Imam Khomeini Hospital, Mazandaran University of Medical Sciences, Sari, IR Iran; 3 Department of Oncology, Imam Khomeini Hospital, Mazandaran University of Medical Sciences, Sari, IR Iran; 4 Department of Internal Medicine, Imam Khomeini Hospital, Mazandaran University of Medical Sciences, Sari, IR Iran

**Keywords:** Renal Dialysis, Anemia, Iron-Deficiency, Ascorbic Acid

## Abstract

**Background:**

Functional iron deficiency (FID) may cause erythropoietin resistance in patients under hemodialysis (HD). Since the role of chronic inflammation or oxidative stress in its pathogenesis is unclear, controversy remains to whether intravenous iron or intravenous ascorbic acid (an antioxidant) can improve this anemia due to decreased iron availability.

**Objectives:**

The current study compared the effect of intravenous iron versus intravenous ascorbic acid in the management of FID in HD patients.

**Patients and Methods:**

Forty HD patients with hemoglobin (Hb) ≤ 11 g/dL, serum ferritin ≥ 500 ng/mL and transferrin saturation (TSAT) ≤ 25% were randomly divided into two groups. 20 patients received 100 mg of intravenous (IV) iron (group I), and 20 patients received 300 mg of IV ascorbic acid (group II) postdialysis, twice a week for 5 consecutive weeks. Hb and iron metabolism indices were measured before the onset of the study and after 12 weeks following therapy.

**Results:**

Twenty one percent of all HD patients, exhibited high serum ferritin, low TSAT and sufficient data for analysis. Both Group I (n = 20) and Group II (n = 20) patients showed a significant increase in Hb, serum iron, and TSAT (P < 0.001). There were no significant differences between both groups in increasing Hb (P = 0.076), serum iron (P = 0.589), serum ferritin (0.725), and TSAT (P = 0.887).

**Conclusions:**

This study showed that both IV iron and IV ascorbic acid can improve FID in HD patients. A larger randomized trial is warranted to determine the optimal management of FID in HD patients.

## 1. Background

In HD patients, an erythropoiesis stimulating agent (ESA) and sufficient iron stores are necessary to effectively produce red blood cells (1). Iron deficiency can occur in HD patients due to continuing blood loss in the dialysis circuit, laboratory testing, interventional procedures and gastrointestinal bleeding (2).

Serum ferritin and transferrin saturation (TSAT) are the laboratory tests which help to diagnose iron deficiency and guide iron therapy in HD patients. However, ferritin and TSAT are positive and negative acute phase reactants, respectively (3). Presence of inflammation in HD patients could be due to various reasons, including acute or chronic infections, underlying autoimmune diseases and malignancies. During the inflammatory process and malnutrition, serum ferritin level increase and TSAT level decrease are difficult to evaluate (4).

In FID, there is improper response to ESA due to failure of iron utilization even with adequate iron stores. It is characterized by high serum ferritin concentration and low transferrin saturation in HD patients (5). There is not any practice guidelines for iron management in HD patients that are most likely to have FID.

Intravenous ascorbic acid in several studies improved responsiveness to ESA in HD patients with refractory anemia and sufficient iron stores possibly by augmenting iron mobilization and antioxidant effect (6, 7).

## 2. Objectives

The aim of this study was to compare the efficacy of intravenous iron sucrose with intravenous ascorbic acid in improving anemia in HD patients with FID.

## 3. Patients and Methods

### 3.1. Patients Population

The present study was a randomized, controlled multicenter trial conducted in 3 medical centers in Mazandaran province, North of Iran from 2010 to 2012. An open label prospective study of three month duration was performed in 40 of 182 patients in three hemodialysis centers. Inclusion criteria were: HD patients ≥ 18 years old, HD therapy for ≥ 3 months, duration of recombinant human erythropoietin (rHu Epo) treatment ≥ 6 months at least 6000 U/week, stable Hb level for four consecutive weeks, average three months Hb level < 11 g/dL, serum ferritin level ≥ 500 ng/mL, and TSAT ≤ 25%. The patients were excluded if they had any of the following events known for the rHuEpo hypo responsiveness such as blood transfusion, blood loss, acute infection, chronic inflammatory diseases, active liver disease, hemoglobinopathy such as sickle cell disease, and treatment with ACE inhibitors. All patients that participated in our study were treated with HD for 4 hours thrice a week, blood flow rate of 250-300 mL/min, and dialysate flow rate of 500 ml/min. Moreover, all patients received folic acid, vitamin B12, and rHu Epo three times a week during the study. The study was approved by the local ethics committee, and all study patients provided informed consent before the start of study.

### 3.2. Data Collection

In the current study, 40 patients were randomly divided (17 men and 23 women) into two groups. In group I, 20 patients (9 men and 11 women) received IV infusion of 100 mg iron sucrose (Venofor st. Gallen/Switzerland) diluted in 100 mL of normal saline, postdialysis, twice a week, during the first 5 weeks of the study.

In group II, 20 patients (8 men and 12 women) received 300 mg IV ascorbic acid postdialysis, twice a week for 5 consecutive weeks. The follow-up period was 12 weeks.

Blood samples were drawn before HD. Hb, serum iron, ferritin, total iron binding capacity (TIBC), and TSAT were measured before the start of study as baseline and at the end of study after 12 weeks. Hb was measured by a computerized coulter counter (Abbot Machine made in USA), serum iron and TIBC were measured by Pars Azmoon kit (made in Iran) and Hitachi analyzer (Model 911, made in Japan), and serum ferritin was measured by Liazon kit (made in Italy). TSAT was calculated by dividing serum iron by TIBC x 100.

### 3.3. Statistical Analysis

Statistical analysis was performed by SPSS software version 16.0 (SPSS Inc, Chicago IL, USA). Comparison of baseline continuous descriptive variables of the two groups was analyzed using Mann-Whitney tests. Paired T-Test was used for comparison of within –group differences between baseline and post treatment period. Frequency distribution was analyzed using Chi square test. P value < 0.05 was considered statistically significant.

In this study the primary outcome was to compare two groups in change of Hb from baseline to week 12. Secondary outcome included a comparison of two groups in change of serum ferritin, serum iron, TIBC, and TSAT from baseline to week 12.

## 4. Results

Twenty one percent of all HD patients were classified as having high serum ferritin and low TSAT. These patients were divided into two groups. Group I included 20 patients who received iron sucrose and group II included 20 patients who received ascorbic acid. The two groups were similar regarding age, sex, duration of hemodialysis, and etiology of end-stage renal disease (Table 1). 

**Table 1.** Demographics of Patients Under Hemodialysis

	Group I (n = 20) (Iron Sucrose)	Group II (n = 20) (Ascorbic Acid)	P value
**Age, y**	60.1 (20.3)	57.1 (15.1)	0.60
**Sex, Male /Female **	9/11	8/12	0.56
**Duration of hemodialysis (Mon), NO. (%)**	28 (2)	26 (1)	0.64
**Type 2 diabetes, NO. (%)**	10 (50)	8 (40)	0.37
**Hypertension, NO. (%)**	4 (20)	6 (30)	0.48
**Glomerular disease, NO. (%)**	2 (10)	3 (15)	0.58
**Other, NO. (%)**	4 (20)	3 (15)	0.54

Table 2 compares hematologic laboratory results at the baseline and after 12-week endpoint in two groups.

**Table 2.** Hematologic Laboratory Results at the Baseline and After 12-Week End Point

Lab Test	Group I (n = 20) (Iron Sucrose)	Group II (n = 20) (Ascorbic Acid)	P value
**Baseline Hb (g/dL)**	9.58 (1.20)	8.43 (1.69)	0.098
**Endpoint Hb (g/dL)**	10.59 (1.23)	9.97 (1.52)	0.242
**Baseline serum ferritin (ng/mL)**	543.85 (233.99)	651.77 (188.07)	0.994
**Endpoint serum ferritin (ng/mL)**	442.66 (271.86)	513.24 (310.58)	0.256
**Baseline serum iron (ng/mL)**	46.85 (23.62)	61.00 (38.26)	0.127
**Endpoint serum iron (ng/mL)**	161.00 (111.18)	156.40 (110.27)	0.948
**Baseline TIBC** ^**a**^ **(µg/dL)**	337.55 (83.54)	317.65 (138.89)	0.328
**Endpoint TIBC (µg/dL)**	500.85 (236.79)	405.75 (179.55)	0.109
**Baseline TSAT** ^**a**^ **(%)**	14.61 (7.17)	18.81 (6.50)	0.719
**Endpoint TSAT (%)**	31.05 (17.23)	34.55 (15.30)	0.640

^a^ Abbreviations: TIBC, total iron binding capacity; TSAT, transferrin saturation

Hb, serum ferritin, Serum iron, and TSAT were similar in two groups at the baseline and endpoint after 12 weeks.

Hematologic variables at the baseline and 12-week endpoint in each group are summarized in Table 3.

**Table 3.** Hematologic Variables at the Baseline and Endpoint in Each Group

Lab Test	Group I (n = 20)			Group II (n = 20)			P value Between 2 Groups
	Baseline	Endpoint	P value	Baseline	Endpoint	P value	
**Hb (g/dL)**	9.58 (1.20)	10.59 (1.23)	< 0.001	8.43 (1.69)	9.97 (1.52)	< 0.001	0.076
**Serum ferritin (ng/mL)**	543.85 (233.99)	442.66 (271.96)	0.182	651.77 (188.67)	513.24 (310.58)	0.085	0.725
**Serum Iron (ng/mL)**	46.85 (23.62)	161.00 (111.18)	< 0.001	61.00 (38.26)	156.40 (110.27)	0.001	0.589
**TIBC (µg/dL)** ^**a**^	337.55 (83.54)	500.85 (236.79)	0.003	317.65 (138.89)	405.75 (179.55)	0.079	0.274
**TSAT** ^**a**^ **(%)**	14.61 (7.17)	31.05 (17.23)	< 0.001	18.81 (6.50)	34.55 (15.30)	0.001	0.887

^a^ Abbreviations: TIBC, total iron binding capacity; TSAT, transferrin saturation

Both groups of patients showed significant increase in Hb, serum Iron, and TSAT during the 12-week analysis period (P < 0.001). Moreover, Group I patients showed significant increase in TIBC (P = 0.003); however, group II patients did not show significant increase in TIBC (P = 0.079). No significant changes in serum ferritin levels were observed in each group during the 12 week analysis period.

There were not any significant differences between the two groups regarding changes in Hb (P = 0.076), serum ferritin (P = 0.725), serum iron (P = 0.589), TIBC (P = 0.274), and TSAT (P = 0.887). As well, there were not any significant differences between the two genders in response to treatment in both groups.

## 5. Discussion

The results of the study showed that administration of intravenous iron sucrose was equally effective as administration of intravenous ascorbic acid in the treatment of functional iron deficiency in HD patients. Both drugs significantly increased Hb, serum Iron, and TSAT in these patients. Anemia in HD patients is associated with several complications such as, increased risk of cardiovascular diseases, decreased quality of life, and overall poor prognosis (8, 9). Therefore, these drugs with correction of anemia are effective in increasing survival of HD patients.

Functional iron deficiency anemia is reported increasingly in HD patients (10). It seems that defective iron mobilization from iron stores and inadequate iron utilization are important mechanisms of ESA hypo responsiveness in these patients (11). KDOQI anemia guidelines published in 2006 specified a lack of sufficient evidence to support the routine use of iron supplement in most HD patients with serum ferritin > 500 ng/mL who were treated with ESAs (2). However, the dialysis patients' Response to IV iron with Elevated ferritin (DRIVE) study demonstrated that intravenous ferric gluconate was superior to no iron in treating anemia in HD patients with ferritin of 500-1200 ng/mL and TSAT ≤ 25% (12). In addition, the DRIVE–II study results showed that administration of 1 g of intravenous gluconate significantly reduced epoetin dose at 12th week in the above patients (13).

Over the 12 week of the DRIVE–II study, there was less risk of hospitalizations from infections among patients given intravenous iron compared to the control group (13). However, almost 50% of patients in DRIVE study were black and patient responsiveness to ESAs and intravenous iron could be due to racial differences.

In another study in the USA, HD patients with Hb less than 11 g/dL, ferritin between 500 and 1200 ng/mL, and TSAT of less than 25%, receiving intravenous ferric gluconate were more than twice as likely to achieve an Hb response ≥ 2 g/dL compared to patients not receiving iron. This response was regardless of baseline CRP and reticulocyte hemoglobin contents (14).

Keyhanian et al. administered 300mg of intravenous ascorbic acid for 3 months to HD patients with Hb less than 11 g/dL, serum ferritin ≥ 300 ng/mL and TSAT < 30% (6). These investigators observed that intravenous ascorbic acid can improve functional iron deficiency in HD patients with refractory anemia. This effect of ascorbic acid could be by augmenting iron mobilization from its tissue stores or antioxidant effects. Tarng et al. compared efficacy of intravenous iron with intravenous ascorbic acid in HD patients with serum ferritin > 500 ng/mL and TSAT < 30% (15). They observed that intravenous iron therapy cannot resolve functional iron deficiency, while intravenous ascorbic acid significantly increased Hb and TSAT and decreased serum ferritin in these patients. This no response to intravenous iron therapy could be due to low total dose of iron administration (100 mg ferric saccharate postdialysis on five consecutive dialysis sessions), while the present study administered 100 mg iron sucrose postdialysis on ten consecutive dialysis sessions.

Attallah et al. reported that intravenous ascorbic acid with each dialysis session increased Hb and TSAT in anemic HD patients with hyperferritinemia by improving responsiveness to ESA (7). Taji et al. on the other hand, did not show any beneficial effect of intravenous ascorbic acid on anemia in HD patients (16).

In this study, serum ferritin was rather reduced even after IV iron treatment. This discrepancy could be explained as since ferritin is a positive acute phase reactant (3), its serum level increases in the setting of malnutrition (4). Treatment of Iron deficiency anemia following IV iron replacement therapy, improved quality of life, appetite, and nutritional state which decreased serum ferritin.

Finally, this study had some limitations. First, small number of patients with high ferritin and low TSAT levels who were available for analysis and insufficient time for study. Second, since we did not perform bone marrow biopsies to access true iron stores, we could not explain any hypotheses regarding the underlying etiologies that drive ferritin to higher levels. However, others have shown that higher ferritin levels may drive by a combination of inflammation and malnutrition in addition to iron stores (5). In conclusion, this study suggested equal efficacy for intravenous iron and intravenous ascorbic acid for treatment of anemic HD patients with serum ferritin ≥ 500 mg /mL and TSAT ≤ 25%. Our data did not show any significant difference between both drugs for treatment of functional iron deficiency anemia in HD patients. More clinical studies are required to determine the exact serum ferritin and TSAT levels to start treatment for maximal response.
